# Insights into Persian Gulf Beach Sand Mycobiomes: Promises and Challenges in Fungal Diversity

**DOI:** 10.3390/jof11080554

**Published:** 2025-07-26

**Authors:** Abolfazl Saravani, João Brandão, Bahram Ahmadi, Ali Rezaei-Matehkolaei, Mohammad Taghi Hedayati, Mahdi Abastabar, Hossein Zarrinfar, Mojtaba Nabili, Leila Faeli, Javad Javidnia, Shima Parsay, Zahra Abtahian, Maryam Moazeni, Hamid Badali

**Affiliations:** 1 Student Research Committee, Mazandaran University of Medical Sciences, Sari 4815733971, Iran; saravani.itf@gmail.com (A.S.); parsa.19gums@gmail.com (S.P.); 2 Departamento de Saúde Ambiental, Instituto Nacional de Saúde Doutor Ricardo Jorge, Avenida Padre Cruz, 1649-016 Lisbon, Portugal; joao.brandao@insa.min-saude.pt; 3 cE3c—Centre for Ecology, Evolution and Environmental Changes, Faculdade de Ciências da Universidade de Lisboa, Campo Grande 016, 1749-016 Lisboa, Portugal; 4 Department of Medical Laboratory Sciences, Faculty of Paramedical, Bushehr University of Medical Sciences, Bushehr 7518759577, Iran; bahram_sound2005@yahoo.com; 5 Infectious and Tropical Diseases Research Center, Health Research Institute, Ahvaz Jundishapur University of Medical Sciences, Ahvaz 6135715794, Iran; a.r.matehkolaie@gmail.com; 6 Department of Medical Mycology, School of Medicine, Mazandaran University of Medical Sciences, Sari 4815733971, Iran; hedayatimt@gmail.com (M.T.H.); m.abastabar@gmail.com (M.A.); javidniaj@gmail.com (J.J.); 7 Invasive Fungi Research Center, Communicable Diseases Institute, Mazandaran University of Medical Sciences, Sari 4815733971, Iran; 8 Allergy Research Center, Mashhad University of Medical Sciences, Mashhad 1394491388, Iran; h.zarrin@gmail.com; 9 Department of Medical Laboratory Sciences, Sar.C., Islamic Azad University, Sari 48164194, Iran; m.nabili2010@gmail.com; 10 Department of Parasitology and Mycology, School of Medicine, Baqiyatallah University of Medical Sciences, Tehran 1435916471, Iran; faelileila@gmail.com; 11 Clinical Tuberculosis and Epidemiology Research Center, National Research Institute of Tuberculosis and Lung Diseases (NRITLD), Shahid Beheshti University of Medical Sciences, Tehran 1956944413, Iran; abtahiann@yahoo.com; 12 Department of Molecular Microbiology & Immunology, South Texas Center for Emerging Infectious Diseases, The University of Texas at San Antonio, San Antonio, TX 78249, USA

**Keywords:** mycobiome, fungal diversity, *Aspergillus* species, the Persian Gulf

## Abstract

Beach Sand Mycobiome is currently among the most important health challenges for viticulture in the world. Remarkably, the study of fungal communities in coastal beach sand and recreational waters remains underexplored despite their potential implications for human health. This research aimed to assess the prevalence of fungal species and the antifungal susceptibility profiles of fungi recovered from the beaches of the Persian Gulf and the Sea of Oman. Sand and seawater samples from 39 stations distributed within 13 beaches along the coastline were collected between May and July 2023. The grown isolates were identified at the species level based on morphological characteristics and DNA sequencing. Antifungal susceptibility testing was performed according to the Clinical Laboratory Standards Institute guidelines. Of 222 recovered isolates, 206 (92.8%) filamentous fungi and 16 (7.2%) yeast strains were identified. Sand-recovered fungi comprised 82.9%, while water-originated fungi accounted for 17.1%. The DNA sequencing technique categorized 191 isolates into 13 genera and 26 species. The most recovered genus was *Aspergillus* (68.9%), and *Aspergillus terreus sensu stricto* was the commonly identified species (26.14%). Voriconazole was the most effective antifungal drug against *Aspergillus* species. Research on fungal contamination levels at these locations could provide a foundation for establishing regulatory frameworks to diminish fungal risks, thereby enhancing public health protection. The ecological significance of fungal communities in sandy beaches to human infections remains to be explored, and earlier reports in the literature may motivate researchers to focus on detecting this mycobiome in natural environments where further investigation is warranted. Ultimately, our discovery serves as a reminder that much remains to be learned about pathogenic fungi and underscores the need for vigilance in areas where emerging pathogens have not yet been identified.

## 1. Introduction

Like other soil types, beach sand comprises organic materials from microorganisms, plants, animals, human-caused activities, and marine influences [1]. The water activity in beach sand is influenced by extended exposure to ultraviolet radiation and saline compounds [2,3]. These elements collectively enhance the microbial diversity found within beach sand. Traditionally, research has predominantly concentrated on pathogenic bacteria and viruses, particularly those linked to gastrointestinal disorders [4,5]. However, the study of fungal communities in coastal beach sand and recreational waters remains underexplored despite their potential implications for human health. Although the association with human infection through recreational water exposure is unclear, swimming was found to be a risk factor for otomycosis and keratitis while wearing contact lenses [6]. Recently, there has been a growing interest in fungi in beach sand, particularly as particular identified species are known to be associated with conditions such as dermatomycoses, allergic responses, otitis, and deep mycetoma [7]. In 2019, a collaborative working group was established by the International Society for Human and Animal Mycology and the European Confederation for Medical Mycology (ISHAM/ECMM) to initiate a comprehensive international project focused on investigating fungal contamination in beach environments and recreational waters [8]. Recently, the “Mycosands” Working Group, a partnership between ECMM, ESCMID, and ISHAM, conducted research on the occurrence of fungi in sand and water samples from 13 countries, predominantly within Europe [9]. The mycosand of several coastal beaches around the world has been studied, including the Southern Gulf of California in Mexico, Mediterranean Sea beaches, the Gulf of Mexico in the United States, and the sand of an urban beach in Slovenia, among others [10]. However, comprehensive distribution data for coastal fungi remain insufficient in numerous regions worldwide. Consequently, limited information is available regarding the abundance of fungal species along the coastal lines of Iran. It is worth noting that the variety and abundance of medically important fungi in the beach sand and water of the Caspian Sea have also been evaluated previously by the same research team [11]. The southern coastlines of Iran, which extend along the Persian Gulf (PG) and the Sea of Oman, cover 2250 km. This region covers a vast portion of the strategically vital, oil-abundant Middle East area and is connected to open seas such as the Oman Sea [12]. The beaches and shorelines of the southern regions are valued recreational areas in Iran, contributing substantially to the income generated by the tourism industry. Assessing these regions for public health purposes is crucial for the safety of the coastal communities. Major port cities on the PG are Bandar-e Mahshahr, Bushehr, Bandar Lengeh, and Bandar Abbas. The PG’s strategic positioning has historically rendered it a prime area for human development. As human activities have intensified in this sub-region, it has been recognized as one of the planet’s most significantly human-related altered areas [13]. The PG and the Oman Sea represent crucial ecosystems with rich biodiversity and abundant fisheries resources [14]. Accordingly, this study reported the mycological quality of beach sand and recreational water at selected beaches, detailing the variety and abundance of species and the antifungal susceptibility profiles of fungi collected from the PG and the Sea of Oman beach sands.

## 2. Materials and Methods

### 2.1. Sites and Sampling

**Figure 1:**
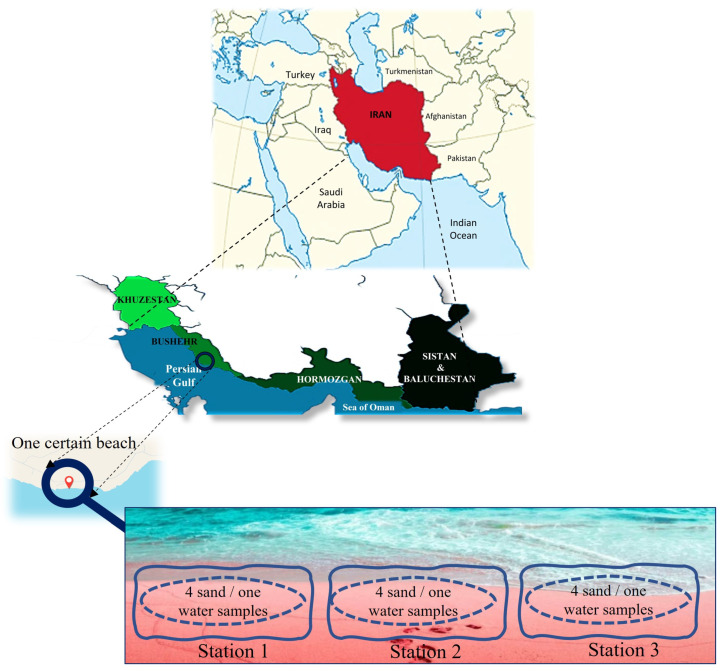
The provinces selected for sampling are located along the coastlines of the Persian Gulf and the Sea of Oman. Each beach was divided into three stations, and from each station, four sand samples were pooled into a single sample, resulting in one sand and one water sample. Finally, 39 sand and 39 water samples were taken from 39 stations. Sampling was performed from sand and water from May to July 2023.

The Persian Gulf and the Oman Sea region come in various shapes and sizes, and have hydrological, ecological, and geological conditions. Across the group, the unifying feature of this diverse landscape is the dominance of water. To ensure accurate dispersal of fungal species along a specific beach, the southern coastal line was divided into four provinces based on geographical divisions. Taking into account the size of each area, population, and potential for tourism attractions, certain beaches were chosen for sampling. The coastline comprises four provinces, including Khuzestan (one urban beach), Hormozgan (4 urban beaches), Bushehr (5 urban beaches), and Sistan and Baluchestan (3 urban beaches) (Figure 1). Each beach was divided into three stations; from each station, four sand samples were pooled into one sample, resulting in one sand and one water sample. Consequently, we had a total of thirteen beaches, resulting in thirty-nine stations (39 sand samples and 39 water samples). However, the primary aim of the study was to analyze the abundance and species distribution of fungi in each province. Some parameters, such as temperature and humidity of the air, were recorded on the sampling day. The measurements were conducted on-site and were not sourced from meteorological data. The beaches were close to one another. Sampling for each province was conducted in a single day. Therefore, the province reported the temperature and humidity as an average. Sampling was performed during the bathing season, involving three sampling rounds. Two locations, comprising Bushehr and Hormozgan, are coastal areas associated with major urban centers and substantial populations. Bushehr and Khuzestan, specifically the port city of Mahshahr, are also recognized as harbor cities involved in the shipping trade. Sistan and Baluchestan are popular beach destinations for tourists across Iran due to their stunning nature. Bathers visit all these beaches during the bathing season, which is typically between late February/beginning of March and late July in the south of Iran. Samplings during the bathing season include three months, i.e., May, June, and July. These sites were chosen due to their extensive coverage of the Persian Gulf Coast, thereby providing valuable insights into the present of fungal flora.

Sand and seawater sampling was performed monthly for each beach in May and July 2023. Considering the tides (rising and falling sea levels), sampling was done between 10.00 am and 1.00 pm at a depth of 5–10 cm as prearranged in the Standard Operating Procedure (SOP) according to Sabino et al. [15] and confirmed by the leaders of the Mycosands project [3]. From each station, almost 200 g of sea sand and 500 mL seawater samples were aseptically collected into sterile 50 mL Falcon flasks and two sterile vessels (Golias, Ljubljana, Slovenia) at a depth of 20 cm in a one-deep water column, respectively. All samples were labeled and transported to the laboratory at 4 °C within two hours of sampling, as described by Sabino et al. [15].

### 2.2. Sample Preparation, Fungal Load, and Phenotypic Identification

Briefly, forty grams of the collected sand were extracted under shaking (100 rpm) with 40 mL of sterile distilled water for 30 min. The sand wash was diluted (1:10), then plated (0.2 mL) on (a) Sabouraud’s dextrose agar (SDA, Madrid, Spain) plates supplemented with chloramphenicol, (b) SDA plates supplemented with chloramphenicol and cycloheximide, (c) SDA plates supplemented with chloramphenicol/itraconazole 4 mg/L; (d) SDA plates supplemented with chloramphenicol/voriconazole 2 mg/L; and (e) SDA plates supplemented with chloramphenicol/posaconazole 0.5 mg/L. Antifungal-supplemented media were utilized for the initial screening and identification of resistant strains. The plates were incubated at 30 °C and monitored for growth up to 3 weeks. Plates containing fluconazole were also incubated at 40 °C for possible isolation of fluconazole-resistant *Candida auris*. The fungal load was evaluated quantitatively by accounting for colony-forming units (CFU)/gram and (CFU)/mL for sand and water, respectively. Sabouraud’s dextrose agar (SDA, Madrid, Spain) plates supplemented with chloramphenicol were applied for reporting CFUs. Phenotypic identification was conducted using mycological techniques, including direct examination (KOH 20%) and culture [3]. Following the growth of fungi on Sabouraud Dextrose Agar (SDA), the cultures were analyzed, and their macroscopic characteristics were recorded. The identification process was further supported by selective media, including CHROMagar (CHROMagar *Candida*, Paris, France) and Czapek agar (HiMedia, India). The microscopic morphology was also assessed through wet mounts stained with lactophenol cotton blue.

#### DNA Extraction, Amplification, and Sequencing

Genomic DNA was extracted from mycelial and yeast cells cultivated for 24–48 h [16], utilizing a phenol/chloroform extraction method. Briefly, a small amount of young colony was transferred to a 2 mL Eppendorf tube containing lysis buffer (200 mM Tris-HCl; pH 7.5), 25 mM EDTA (Ethylenediaminetetraacetic Acid), 0.5% *w*/*v* SDS (Sodium Dodecyl Sulfate), and 250 mM NaCl. The mixture was mechanically crushed using a conical grinder for 3 min. Following this, phenol-chloroform (1:1) was added, after being centrifuged at 12,000 rpm, chloroform was added to the supernatant, followed by another centrifugation at 12,000 rpm. DNA was precipitated by adding 30 μL of sodium acetate and 300 μL of isopropanol. Subsequently, the DNA pellets were diluted with cold 70% ethanol, dried using a heat block, suspended in double-distilled water (DDW), and stored at −20 °C. Molecular Identification was performed by DNA sequencing of *TEF1* (translation elongation factor 1 complex) for *Fusarium* species, *TUB1* (beta-tubulin) and *CMD* (Calmodulin) genes for *Aspergillus* and *Penicillium* species, and the ITS rDNA region for other non-dermatophyte molds, dematiaceous fungi, and yeast species as previously described. The sequences of the primer pairs used for each region’s amplification were as follows: Cmd-F (5′-CCGAGTACAAGGAGGCCTTC-3′), Cmd-R (5′-CCGAGTACAAGGAGGCCTTC-3′), EF1-F (5′-ATG-GGT-AAG-GAG-GAC-AAG-AC-3′), EF1-R (5′-GGA-AGT-ACC-AGT-GAT-CAT-GTT-3′), BT1-F (5′-AAC-ATG-CGT-GAG-ATT-GTA-AGT-3′), and BT1-R (5′-TAG-TGA-CCC-TTG-GCC-CAG-TTG-3′), along with ITS 1 (5′-TCCGTAGGTGAACCTGCGG-3′) and ITS 4 (5′-TCCTCCGCTTATTGATATGC-3′) [17,18,19]. The obtained sequence raw data were aligned manually using MEGA6.05 and BioEdit (Version 7.0.9, Alignment, BioEdit Sequence 2011) software packages. Sequencing was performed on an ABI3730 sequencer (Applied Biosystems, Foster City, CA, USA). Sequence data were adjusted using the SeqMan of Laser gene software (version 9.0.5, DNA Star, Madison, WI, USA) and compared with the GenBank database and submitted to the GenBank database(http://blast.ncbi.nlm.nih.gov/Blast.cgi, accessed on 8 October 2024).

### 2.3. Antifungal Susceptibility Test Assay for Selected Isolates

Antifungal susceptibility tests were performed using the Clinical and Laboratory Standards Institute (CLSI) guidelines M38, M60, and M59 for filamentous and yeast isolates recovered from antifungal-supplemented media. The document M59 was also referred to for antifungal without specific breakpoints [20,21,22]. The antifungal agents were diluted in a standard RPMI 1640 medium (Sigma-Aldrich, St. Louis, MO, USA) and then buffered to pH 7.0 with 0.165 M 3-(N-Morpholino) propanesulfonic acid (MOPS, Sigma-Aldrich, St. Louis, MO, USA.) with L-glutamine without bicarbonate to yield twice their concentration. Subsequently, they were distributed into 96-well microdilution trays (Nunc, UK) with a final concentration of 0.016–16 µg/mL for itraconazole (ITZ), voriconazole (VRZ), and posaconazole (PSZ), 0.063–64 µg/mL for Fluconazole. MICs were read visually at >80% growth inhibition after 24 h of incubation at 35 °C for the tested drugs compared to positive controls. *Candida parapsilosis* (ATCC 22019) and *Hamigera insecticola* (previously identified as *Paecilomyces variotii*) (ATCC 22319) were used as the quality control isolates and were included on each day of testing.

### 2.4. Statistical Analyses

All statistical analyses were performed using SPSS v25 (SPSS Inc., Chicago, IL, USA). Descriptive analysis for parameters was performed, using means and 95% confidence intervals for mean, median, minimum, and maximum values for continuous variables. Student’s t-test was performed on all variables of this study. MIC ranges, MIC50, MIC90, and geometric mean (GM) MICs were calculated.

## 3. Results

### 3.1. Fungal Community Flora in Sand and Seawater

**Figure 2:**
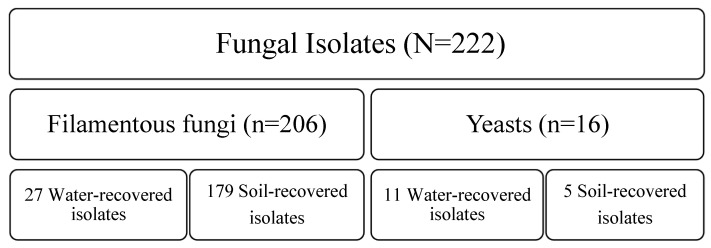
The categorized, detailed results for the fungal community flora in sand and seawater. Of 222 isolates, 206 filamentous fungi and 16 yeasts were recovered. Amongst 38 water-recovered isolates, 27 and 11 isolates were identified as filamentous fungi and yeast, respectively. 184 isolates were recovered from sand, including 179 filamentous fungi and 5 yeast isolates.

**Figure 3:**
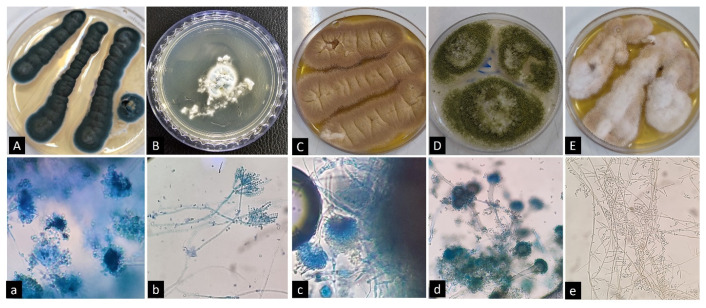
Microscopic and macroscopic features of some fungal species isolated from soil/water samples during May to July, 2023: (**A**,**a**): *Aspergillus sydowii*, (**B**,**b**): *Penicillium chrysogenum*, (**C**,**c**): *Aspergillus terreus*, (**D**,**d**): *Aspergillus flavus/oryzae*, (**E**,**e**): *Fusarium solani*.

Fungal identification, especially concerning environmental fungi such as mold species, presents significant challenges at the species level. Often, reliance on phenotypic characteristics results in the ability to distinguish only the genus, failing to provide clarity regarding specific species. However, the three-month collection of beach sand and seawater samples on the coastline resulted in the phenotypic identification of 222 fungal isolates. Of 222 isolates, 206 filamentous fungi, accounting for approximately 92.8% of the total, and 16 yeasts, representing around 7.2%, were recovered. Various fungal species were found in the beach sands (n: 184, 82.9%). Only 38 strains, comprising 17.1% of the species, were collected from water (Figure 2). Yeast species were predominantly isolated from the Sistan and Baluchestan provinces, presenting 11 out of 16 samples (68.75%); however, the distribution of filamentous fungi was varied according to recovery locations. The distribution was as follows: *Aspergillus* sec. *Terrei* (n: 43, 28.1%), *A.* sec. *Flavi* (n: 39, 25.5%), *A.* sec. *Fumigati* (n: 33, 21.6%), *A.* sec. *Nigri* (n: 37, 24.2%); and other *Aspergillus* species (n: 1, 0.65%). *Fusarium* species was the second common genus (n: 11, 5.34%); Black fungi and *Penicillium* species ranked in third and fourth places with distributions of 10 (4.85%) and 6 (2.91%), respectively. It is important to highlight that the above-mentioned results were evaluated based on the morphological characteristics of the isolated fungi. The precise outcomes were subsequently determined through molecular identification of the samples. No dermatophyte isolates were identified in any of the samples analyzed. Figure 3 illustrates the microscopic and macroscopic features of some fungal species isolated from soil/water samples.

### 3.2. Fungal Load (CFUs) in Sand and Seawater

The first sampling was conducted in May 2023. The temperature ranged from 27 to 29 °C, and the humidity was measured between 51% and 69%. The most humid province was Hormozgan, with a humidity level of 69%. Most yeast species were recovered from Hormozgan, resulting in 65 CFU/mL from water samples incubated at 30 °C. Despite the high humidity of Hormozgan (69%), it is worth noting that most filamentous fungi were recovered from Bushehr, not Hormozgan, resulting in 160 CFU/g, with the predominance of *A.* sec. *Nigri* from sand samples incubated at 30 °C. *Aspergillus* sec. *Fumigati* was recovered as the second most abundant section. Other *Aspergillus* sections were not detected during the first sampling round.

The second sampling occurred in June, when the humidity and temperature increased to 60–68% and 35–39 °C, respectively. For a second time, the most humid province was Hormozgan, with a humidity level of 68%. Remarkably, the majority of filamentous fungi were recovered from Bushehr, resulting in 382 CFU/g, with the predominance of Black fungi (68 CFU/g), followed by *A.* sec. *Terrei* (47 CFU/g), *A.* sec. *Flavi*, *A.* sec. *Fumigati* and *A.* sec. *Nigri* isolated from sand samples incubated at 30 °C. Surprisingly, the number of fungal species capable of tolerating higher temperatures (≥40 °C) increased significantly. Sistan and Baluchestan showed the majority of yeast species from water samples incubated at 30 °C (55 CFU/mL).

**Figure 4:**
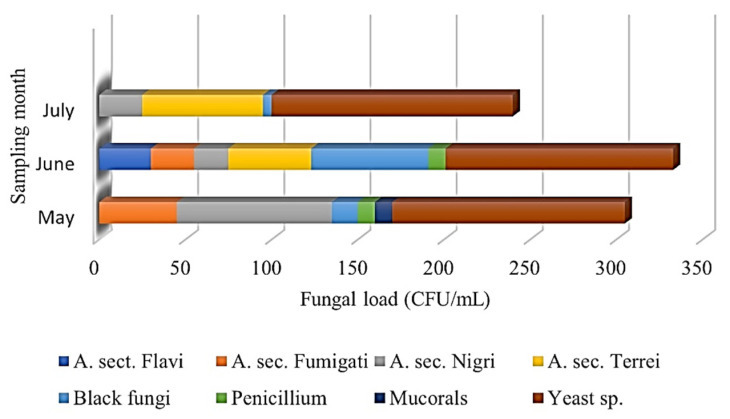
The abundance of fungal species isolated from beach sand based on morphological data. The most diverse fungal species were recovered during June, with the highest CFUs for Yeast species, Black fungi, and *A.* sec. *Terrei*.

During the third round of sampling in July, the temperature and humidity rose to 40–42 °C and 57–71%, respectively. Although Hormozgan was the warmest and most humid province for the third time, high volumes of fungi were recovered from Bushehr with a sand fungal community of 145 CFU/mL, predominantly consisting of *A.* sec. *Terrei* (55 CFU/g) and *A.* sec. *Nigri* (10 CFU/g). Conversely, most yeast species were recovered from Hormozgan once more, yielding 125 CFU/mL from water samples incubated at 30 °C. The data indicated that Bushehr had the highest fungal diversity in the sand. At the same time, in terms of water, most yeast species were reported from Hormozgan, which experienced the highest humidity during the three sampling rounds. Figure 4 illustrates the prevalence of fungal diversity collected from beach sand and the sampling timing. Moreover, the detailed findings for the fungal load (CFUs) are depicted in Table 1.

### 3.3. Molecular Identification of Fungal Isolates

A 3-month survey with a monthly sampling of beach sand provided insights into the diversity of culturable fungal species constituting the fungal community. Out of 222 recovered isolates, 191 (86.03%) were successfully identified at the species level by DNA sequencing. Of the 191 recovered isolates from beach sand/water, 13 genera and 26 different species were categorized. The genus *Aspergillus* revealed 153 (80.1%) isolates distributed in 10 species, comprising: *A.* sec. *Terrei* (*A. terreus*; n: 40, 26.14%), *A.* section *Flavi* (*A. flavus/oryzae*; n: 34, 22.22%); *A.* sec. *Nigri* (*A. niger*; n: 22, 14.38%), *A. tubingensis* (n: 15, 9.80%), *A. neoterreus* (n: 3,1.96%), *A.* sec. Fumigati (*A. fumigatus* (n: 26, 16.99%), *A. parasiticus* (n: 2, 1.31%), *A. nomius* (n: 3,1.96%), *A.* sec. Nidulantes (*A. unguis* (n: 1, 0.65%), *A. sydowii* (n: 7, 4.57%).

**Figure 5:**
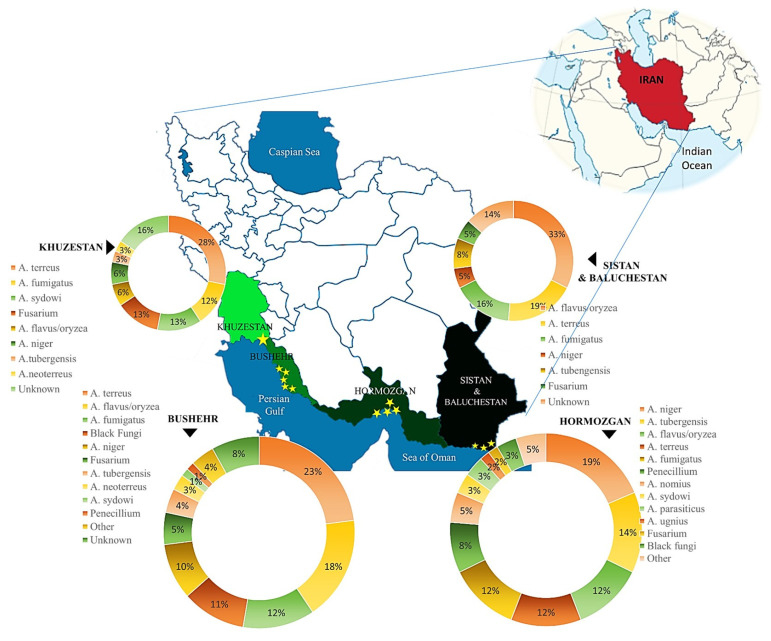
The detailed findings for fungal species distributed across the studied provinces as revealed during the collection of strains for the entire period from May to July 2023.

Among the *Aspergillus* genus, which comprised the most prevalent fungi, *A. terreus* was the predominant *Aspergillus* species isolated during June and July, followed by species of sec. *Fumigati* (only *A. fumigatus*) and species of the sec. *Flavi* (mostly *A. flavus/oryzae*). Notably, these results varied depending on the province from which the fungi were isolated (*A. niger* and *A. flavus/oryzae* were reported as the common *Aspergillus* species from Hormozgan and Sistan-Baluchestan provinces, respectively). The most diverse fungal species were collected from Hormozgan, with mean air temperatures and humidity levels of 35.6 °C and 69.3%, respectively. This result was particularly notable during the first month of sampling in May. *Fusarium* species was the second common genus (n: 11, 5.76%), amongst *F. incarnatum-equiseti* species complex (n: 5, 45.45%), *F. Sarocladium* (n: 4, 36.36%), and *F. solani* (n: 2, 18.18%). *Penicillium* (mainly *P. citrinum)* and black fungi ranked third and fourth place, with distributions of 6 (3.14%) and 5 (2.68%), respectively. The *Candida* species represented 56.25% of the yeast isolates, including *C. parapsilosis* (n: 6/16, 37.5%) and *Nakaseomyces glabratus* (*C. glabrata*) (n: 3/16, 18.75%). Remarkably, the presence of *Rhodotorula* (n: 7/16, 43.75%) in sand samples makes it the second most prevalent yeast isolate. Table 2 and Figure 5 depict the detailed findings for fungal diversity across the studied provinces.

### 3.4. Antifungal Susceptibility Test Assay:

Antifungal susceptibility testing was conducted only on the isolates obtained at least once from SDA supplemented with itraconazole, voriconazole, and posaconazole. The grown isolates are probably resistant to the antifungal agents; however, the AFST assay was performed to validate these findings. Among the 22 non-*Aspergillus* filamentous fungi, 14, 2, and 3 strains showed MIC values of >32 µg/mL for itraconazole, voriconazole, and posaconazole, respectively. Two *P. citrinum* isolates showed MIC values of ≥32 µg/mL against itraconazole and voriconazole, except for one *P. citrinum* strain, which exhibited high MIC values of 8 µg/mL against voriconazole (Table 3). Although *A. tubingensis* isolates are intrinsically resistant to itraconazole, *A. terreus* (5/40, 12.5%) isolated from Sistan-Baluchestan sand samples revealed the highest MIC value for itraconazole as resistant isolates according to the CLSI M59 [22]. Table 4 shows the MIC values for all screened *Aspergillus* species grown on antifungal-supplemented SDA media. Table 5 depicts the AFST results for all recovered yeast species from SDA without antifungals.

## 4. Discussion

The present study aimed to improve the awareness of sand and seawater mycobiota by analyzing fungal diversity. Furthermore, it investigated the monthly fluctuations of the dominant species during the bathing season. The data presented here prompts an important question regarding assessing the potential impact of environmental fungi on our health. Since many of the fungal species found in sands are recognized as potential allergens and opportunistic pathogens in humans, these findings may represent a possible environmental source of infection in otherwise healthy individuals (i.e., asthmatic patients, COPD, cystic fibrosis patients). Therefore, addressing this issue needs complicated epidemiological assessments. Understanding whether there is a rise in fungal infections and allergic conditions among both immunocompromised and immunocompetent individuals who frequently visit these beaches, compared to those from other regions, is essential. This relationship has been previously explored in other pathology/context binomials, such as gyms and dermatophytes, and building sickness [23,24]. Another significant concern to consider is the ascending trend in antifungal resistance. In recent years, the impact of human activities and climate change on the patterns of seawater mycobiome has emerged as a significant global concern [25]. Although the activities of beach visitors and swimmers can undoubtedly influence the sand microbiome, various species may also be introduced to the sandy coastline through water tides that transport them from the depths of the seawater to either the wet or dry areas of the shore. The autochthonous microbiota found on sandy beaches can be influenced by microbial deposition, coastal erosion, and the rise of antimicrobial resistance [26]. Furthermore, global warming, increasing human populations, and climate change are anticipated to impact both the diversity and quantity of microbiota, including mycobiota [3,27]. Global warming and heat waves, in particular, have been predicted to generate selective pressure that will lead to the emergence of new pathogens due to higher temperature tolerances [28]. Alterations in air and water temperature, hours of sunshine, humidity and precipitation, air pressure, and wind speed seem to influence mycobiota [7]. For the first time in Iran, this study provided data on the fungal content of sand and water along the PG coasts during the bathing season in southern Iran. Our study revealed that potentially harmful fungi could be extracted from the sand and water at the beaches, with species belonging to the genus *Aspergillus* representing the most significant portion of the identified fungal species. *Aspergillus terreus* has been described as the predominant *Aspergillus* species. The *A. terreus* species is particularly noteworthy due to its typical resistance to amphotericin B and its distinct immune interaction characteristics, which result in worse outcomes than *A. fumigatus* [29,30,31]. In Iran, *A.* sec. *Flavi* has been reported as the primary etiological fungal agent causing either colonization or allergic/infectious respiratory diseases [32,33]. Moreover, the *Flavi* section was the predominant *Aspergillus* section among fungi recovered from the Caspian Sea coastline [14]. It is noteworthy that *A. terreus* is the most abundant species in the south of Iran. The variation in the dominant species can be primarily attributed to the differing climates of the regions, even though both are situated along coastal areas. The PG coastline is in a subtropical, hyper-arid region, typically marked by elevated temperatures and minimal rainfall. Mediterranean systems predominantly influence the climate in this area, although the eastern sections are affected by the Indian Ocean. The annual temperature and precipitation levels in the PG region fluctuate considerably, primarily determined by geographical and orographic factors. On the southern coasts and over the Gulf, the average annual rainfall is below 100 mm, whereas the northern regions experience an average of 355 mm of precipitation yearly [34]. On the other hand, several reports identified *A. terreus* as the causative agent of invasive pulmonary aspergillosis [30,35,36]. Therefore, persons exposed to high amounts of conidia may have an enhanced risk of developing respiratory symptoms. In the other research, the analysis of the distribution and diversity of all identified genera over 12 months at an Urban Beach in Slovenia revealed that 10 species, specifically *Actinomucor elegans, A. flavus, A. lentulus, A. terreus, Candida tropicalis, Fusarium solani, Microascus brevicaulis, Phialophora americana, Sarocladium kiliense*, and *Trichosporon asahii,* were classified as microorganisms of Biosafety Level 2. Amongst the collected isolates, *Aspergillus* spp. were the most abundant [7].

Generally, increased trends in emerging azole-resistant *Aspergillus* species make disease management more complicated [19,37,38,39]. Our findings indicate that 13.1% of the strains, including intrinsic-ITZ-resistant *A. tubingensis*, were identified as ITZ-resistant. If *A. tubingensis* was excluded, the percentage decreased to 6.53%. In the case of VRZ and PSZ, 1.3% and 6.53% of *Aspergillus* strains exhibited elevated MICs against these antifungals, respectively. The *Fusarium* genus, recognized as the second most frequently isolated filamentous fungi, demonstrated a resistance rate of 36.36% to ITZ. Although *Penicillium* species constituted only 3.14% of the identified isolates, 83.3% of these species displayed high MIC values against ITZ. Notably, dermatophyte species were not isolated in this study, likely due to the absence of hair-baiting techniques for their collection. Nevertheless, whether linked to humans or animals, dermatophyte contamination poses a considerable risk, particularly for individuals engaged in sand-related activities, heightening the likelihood of conidia contact and transmission. Therefore, the health quality of beaches should be regarded as a critical aspect of public health protection, as emphasized in the recent WHO guidelines and highlighted in the WHO’s Fungal Priority Pathogens list to inform research, development, and public health initiatives [40]. Quantitative microbial risk assessment has been applied to estimate health risks from exposure to beach sand [41]. Sabino et al. recommended maximum levels of 15 CFU/g for yeasts, 17 CFU/g for potential pathogenic fungi, and 8 CFU/g for dermatophytes [15,42] on beach sand. All beaches revealed high CFUs for yeast during May and June (>15 CFU/g). Except for Hormozgan, the yeast load diminished to under 15 CFU/g during July (see Table 1). The yielded fungal load for filamentous fungi was also more than the maximum levels of 17 CFU/g. Consequently, research on fungal contamination levels at these locations could provide a foundation for establishing regulatory frameworks to diminish fungal risks, thereby enhancing public health outcomes. Additionally, there are currently no regulations addressing fungal contamination at beaches. Therefore, disinfection of sand is of great concern for appropriate coastal management practices. The primary challenge ahead will be to analyze the mycobiome of the coastline across more extensive beaches, including islands like Kish and Gheshm, which are particularly attractive to tourists. Furthermore, it is highly recommended to conduct genotyping studies to better understand the relationships among fungal species. Another constraint of this research was the sampling duration, which could be prolonged in future bathing seasons, typically regarded from October to December.

## 5. Conclusions

Among *Aspergillus* species, which accounted for 80.1% of the recovered isolates, amphotericin B-resistant *A. terreus* was reported as the predominant filamentous fungus along the studied coastline. Moreover, *A. fumigatus* and *A. flavus/oryzae* were recognized and identified as the species with the highest fungal load during the first and second rounds of sampling (in June), when most beachgoers visit the coastline areas at that time. According to the recently published WHO document, the species *A. fumigatus* is classified as a critical-priority fungal group. Also, *C. parapsilosis*, *N. glabratus,* and *Fusarium* are classified as high-priority fungal groups [40]. Although these agents are frequently present in the environment, repeated exposure could pose a risk, given the importance of the Persian Gulf coast for recreational activities in Iran. Interestingly, 43.75% of the grown yeasts were identified as *Rhodotorula* species, which could now be a significant concern [34]. Therefore, increasing swimmers’ awareness of simple hygiene procedures is essential. Developing standardized sanitation programs, raising awareness among beach users, and establishing criteria for assessing public health risks are crucial elements in the health management of these areas. Extensive studies will help understand the microbial community and their interactions might be a key point for selecting microbial strains able to cause diseases. Although viable *C. auris* was not detected in the coastal habitat in the Persian Gulf, the ecological significance of this multidrug-resistant yeast in salt marsh wetlands and sandy beaches to human infections remains to be explored.

## Figures and Tables

**Table 1:** Fungal load (CFU/mL or CFU/g) based on both sampling rounds and sampling locations during May to July 2023.

**PROVINCE**		**Sample Type**	**Fungal load (CFUs) (30 °C)**																		**First Round (May)**
**Temperature (°C)**	**Humidity (%)**		***A.* Section *Flavi***		***A.* section *Fumigati***		***A.* section *Nigri***		***A.* section *Terrei***		**Black fungi**		***Penicillium* sp.**		**Mucorales**		**Yeast sp.**		**Other**		
Khuzestan		Sand					10														
29	55	Water															30				
Bushehr		Sand			25		75						5				5		55		
28	51	Water													5				25		
Hormozgan		Sand			10												15		40		
27	69	Water			5						15				5		65		40		
Sistan-Baluchestan		Sand			5								5				20		20		
28	52	Water																			
**PROVINCE**		**Sample type**	**Fungal load (CFUs) (30 °C)**																		**Second Round (June)**
**Temperature (°C)**	**Humidity (%)**		***A.* Section *Flavi***		***A.* section *Fumigati***		***A.* section *Nigri***		***A.* section *Terrei***		**Black fungi**		***Penicillium* sp.**		**Mucorales**		**Yeast sp.**		**Other**		
Khuzestan		Sand			10		5												5		
37	62	Water	5		10		5										25		5		
Bushehr		Sand	10		5		10		33		68		5				47		122		
35	60	Water							5				5								
Hormozgan		Sand	5																25		
38	68	Water	5																		
Sistan-Baluchestan		Sand	5						5								15		60		
39	66	Water							5								45		5		
**PROVINCE**		**Sample type**	**Fungal load (CFUs) (30 °C)**																		**Third Round (July)**
**Temperature (°C)**	**Humidity (%)**		***A.* Section *Flavi***		***A.* section *Fumigati***		***A.* section *Nigri***		***A.* section *Terrei***		**Black fungi**		***Penicillium* sp.**		**Mucorales**		**Yeast sp.**		**Other**		
Khuzestan		Sand											10						115		
41	59	Water																	25		
Bushehr		Sand					10		55								10		70		
40	57	Water																			
Hormozgan		Sand					15		15		5										
42	71	Water															125				
Sistan-Baluchestan		Sand															5		15		
41	61	Water																			

**Table 2:** The abundance of fungal species identified by the DNA-sequencing technique during May to July 2023.

Province	Fungal Genus	Fungal Species	Number of Isolates
Khuzestan	*Aspergillus*	*flavus/oryzae*	2
		*terreus*	9
		*fumigatus*	4
		*niger*	2
		*tubingensis*	1
		*neoterreus*	1
		*sydowii*	4
	*Fusarium*	*sarocladium*	1
		*incarnatum-equiseti* species complex	3
	Yeasts	*C. parapsilosis*	2
		*Rhodotorula*	2
	Unknown		5
Bushehr	*Penicillium*	*citrinum*	1
	*Aspergillus*	*flavus/oryzae*	13
		*terreus*	17
		*fumigatus*	9
		*niger*	7
		*tubingensis*	3
		*neoterreus*	2
		*sydowii*	1
	*Fusarium*	*solani*	2
		*sarocladium*	1
		*incarnatum-equiseti species complex*	1
	Black fungi	*Alternaria alternata*	3
	Other	*Lecanicillium psalliotae*	1
		*Apiospora marii*	1
		*Scopolariopsis brevicaulis*	1
	Unknown		11
Hormozgan	*Penicillium*	*citrinum*	3
		*chrysogenom*	2
	*Aspergillus*	*flavus/oryzae*	7
		*terreus*	7
		*fumigatus*	7
		*niger*	11
		*tubingensis*	8
		*unguis*	1
		*sydowii*	2
		*nomius*	3
		*parasiticus*	2
	*Fusarium*	*sarocladium*	1
	Black fungi	*Stachybotrys chartarum*	1
		*Epicoccum*	1
	Other	*Trichoderma asperellum*	1
		*Simplicillium species*	2
	Unknown		4
Sistan and Baluchestan	*Aspergillus*	*flavus/oryzae*	12
		*terreus*	7
		*fumigatus*	6
		*niger*	2
		*tubingensis*	3
	*Fusarium*	*sarocladium*	1
		*incarnatum-equiseti species complex*	1
	Yeast	*C. parapsilosis*	4
		*N. glabratus*	3
		*Rhodotorula*	5
	Unknown		5
Total	*Aspergillus*		153
	*Penicillium*		6
	*Fusarium*		11
	Black fungi		5
	Yeasts		16
	Unknown		31

**Table 3:** The MIC values for all screened non-*Aspergillus* species recovered from antifungal-supplemented SDA media from water/sand samples during the whole period of sampling.

Isolates	Fungal Species	MIC (µg/mL)		
		*ITZ	^£^VRZ	^¥^PSZ
*Penicillium*	*P. citrinum* (4)	≥32	≥32	0.063
		≥32	≥32	0.032
		≥32	8	0.25
		0.25	0.25	0.032
	Other *Penicillium sp.* (2)	≥32	0.5	0.25
		≥32	2	0.25
*Fusarium*	*F. solani* (1)	≥32	1	≥32
	*F. sarocladium* (2)	0.063	0.125	0.032
		≥32	0.25	≥32
	*F. incarnatum-equiseti species complex* (3)	0.032	0.032	0.032
		≥32	0.25	≥32
		≥32	1	1
*Scopulariopsis brevicaulis* (1)		0.032	0.125	0.032
*Trichoderma asperellum* (1)		≥32	1	1
*Simplicillium sp.* (2)		≥32	0.125	0.032
		≥32	0.25	0.032
*Chrysogenum* (2)		0.063	0.125	0.063
		0.032	0.25	0.032
Black fungi	*Epicoccum* (1)	≥32	0.25	0.032
	Other black fungi (3)	≥32	0.125	0.032
		2	1	0.032
		0.25	0.063	0.032

*ITZ: itraconazole; ^£^VRZ: voriconazole; ^¥^PSZ: posaconazole.

**Table 4:** The AFST results for all screened *Aspergillus* species recovered from antifungal-supplemented SDA media from water/sand samples during the whole period of sampling.

*Aspergillus* Species (n)	Number of Isolates		Antifungal Agent	MIC (µg/mL)											MIC Range	MIC_50_	MIC_90_	GM
	Drug Susceptible	Drug Resistant		32	16	8	4	2	1	0.5	0.25	0.125	0.062	0.031				
*Aspergillus fumigatus* (11)	10	1	*VRZ						1	2	2	4		2	0.031-1	0.5	1	0.6299
	9	2	^£^ITZ	2						2	1	1	1	4	0.031->32	0.031	32	0.6382
	10	1	^¥^PSZ	1					1	1	4		1	3	0.031->32	0.031	0.032	0.032
*Aspergillus flavus/oryzae* (21)	21	-	VRZ						1		8	11	1		0.062-1	0.25	1	0.3968
	20	1	ITZ	1							1	5	9	5	0.031-32	0.031	0.25	0.032
	20	1	PSZ							1	2		3	15	0.031-32	0.031	0.25	0.0505
*Aspergillus terreus* (27)	27	-	VRZ						3	9	7	8			0.125-1	0.5	1	0.6040
	22	5	ITZ	5					2	4	9			7	0.031-32	0.5	1	0.3314
	25	2	PSZ	2						5		3	2	15	0.031-32	0.5	0.5	0.1473
*Aspergillus tubingensis* (15)	14	1	VRZ	1						6	1	4		3	0.25-1	0.5	0.5	0.5358
	-	15	ITZ	15											32	32	32	32
	14	1	PSZ						1	10	4				0.031-32	0.5	0.5	0.251
*Aspergillus niger* (5)	5	-	VRZ						2	2	1				0.25-1	0.75	1	0.7071
	4	1	ITZ	1							1	1	1	1	0.031-32	0.063	25	0.4010
	5	-	PSZ						1					4	0.031-1	0.032	1	0.075
*Aspergillus sydowii* (3)	3	-	VRZ							2		1			-	-	-	-
	2	1	ITZ	1						1			1		-	-	-	-
	3	-	PSZ								1			2	-	-	-	-
*Aspergillus unguis* (1)	1	-	VRZ										1		-	-	-	-
	1	-	ITZ										1		-	-	-	-
	1	-	PSZ											1	-	-	-	-

*ITZ: itraconazole; ^£^VRZ: voriconazole; ^¥^PSZ: posaconazole.

**Table 5:** The AFST results for all recovered yeast species from SDA without antifungals from water/sand samples during the whole period of sampling.

Yeast Species	MIC (µg/mL)			
	*FLZ	**ITZ	^£^VRZ	^¥^PSZ
*C. parapsilosis*	0.5	0.25	0.063	0.032
	1	0.25	0.063	0.032
	0.25	0.032	0.125	0.032
	0.5	0.125	0.125	0.063
	1	0.125	0.25	0.032
	0.5	0.125	0.25	0.063
*N. glabratus*	8	0.25	0.25	0.063
	16	1	0.25	0.063
	32	1	0.25	0.032
*Rhodotorula sp.*	64	2	1	2
	16	2	1	1
	≥64	4	1	1
	64	4	2	1
	64	4	1	1
	≥64	4	2	2
	≥64	8	2	2

*FLZ: fluconazole; **ITZ: itraconazole; ^£^VRZ: voriconazole; ^¥^PSZ: posaconazole.

## Data Availability

Some or all data related to AFST and species identification that support the findings of this study are available from the corresponding author (M.M.) upon reasonable request.

## Abbreviations

The following abbreviations are used in this manuscript:

CLSI	Clinical & Laboratory Standards Institute
DOAJ	Voriconazole
TLA	Itraconazole
LD	Posaconazole
PG	Persian Gulf
